# Evidence for sustained physiological adaptation between consecutive exercise bouts at simulated altitude

**DOI:** 10.14814/phy2.70195

**Published:** 2025-04-11

**Authors:** Kelsey E. Joyce, M. Travis Byrd, Courtney M. Wheatley‐Guy, Jesse C. Schwartz, Jordan K. Parks, Bruce D. Johnson

**Affiliations:** ^1^ Human Integrative and Environmental Physiology Laboratory Department of Cardiovascular Diseases, Mayo Clinic Scottsdale Arizona USA; ^2^ School of Sport, Exercise and Rehabilitation Sciences University of Birmingham Birmingham UK

**Keywords:** altitude, hypoxia, intermittent hypoxic training

## Abstract

Intermittent hypoxia has been used to enhance oxygen delivery in athletes and patients; however, it is unclear whether acute exposure is sufficient to elicit lasting physiologic adaptation(s). The purpose of this study was to evaluate physiologic response(s) to hypobaric‐hypoxic (HH) exercise. Nine participants (4 M/5F; 37.9 ± 12.7 yrs.; 174.3 ± 9.4 cm; 75.3 ± 15.9 kg; 24.4 ± 3.4 kg/m^2^) were exposed to progressively higher simulated altitudes and completed two HH submaximal exercise sessions (~30 min ea., ≥72 h apart) on a cycle ergometer at the first altitude that posed a significant challenge to them. Altitude was dependent on individual response as determined from heart rate (HR), peripheral oxygenation (SpO_2_), and the ratio of HR response to SpO_2_ (HR/SpO_2_). Statistical analyses included paired samples *t*‐test (*p* ≤ 0.05). No significant change in SpO_2_ (HH‐1: 85 ± 4% vs. HH‐2: 85 ± 4%, *p* = 0.684) was observed between sessions. However, there were significant decreases in: HR (HH‐1: 150 ± 18 bpm vs. HH‐2: 133 ± 27 bpm, *p* = 0.001) of 18 bpm (11%); HR/SpO_2_ (HH‐1: 1.76 ± 0.22 vs. HH‐2: 1.57 ± 0.33, *p* = 0.012); and RPE (HH‐1: 15 ± 2 vs. HH‐2: 11 ± 4, *p* = 0.017). While workload significantly increased (HH‐1: 89 ± 36 W vs. HH‐2: 105 ± 36 W, *p* = 0.024). Some participants had a threshold/challenging altitude, but from a single bout there is evidence of improved tolerance that can last over a week. Further investigation is required to replicate and understand possible mechanisms.

## 1 INTRODUCTION

Repeated exposure to normobaric or hypobaric hypoxia (intermittent and prolonged, active and passive) have been incorporated by athletes into training programs to enhance athletic performance (Wilber, 2004) with a range of mechanisms cited as facilitators (e.g., increased red blood cell number and size, increased erythropoietin production, and improved pH regulation and buffering capacity) (Gore et al., 2001; Lobigs et al., 2018; Wehrlin et al., 2006). Similarly, repeated intermittent hypoxic exposures have been used by sojourners at rest and during exercise prior to ascending to high altitude in order to attenuate altitude‐related illnesses and improve summit success at altitude (Beidleman et al., 2004; Wille et al., 2012). Resting acute intermittent hypoxic exposures have also been used among patient populations (e.g., cardiovascular disease (Serebrovskaya & Xi, 2016), chronic obstructive pulmonary disease (Burtscher et al., 2009), and spinal cord injury (Trumbower et al., 2012)) to improve outcomes/disease status.

The rapid physiologic adjustments, such as, the hypoxic ventilatory response and ventilatory acclimatization to these types of environmental stresses are well established (Donoghue et al., 2005; Teppema & Dahan, 2010). However, it remains unclear whether a single bout of hypobaric hypoxic exercise lasting ~30 min is sufficient to produce evidence of lasting response(s) or adaptation(s) among recreationally active individuals. The purpose of this study was to examine for evidence of physiologic adaptation after repeat bouts of hypoxic exercise at the same simulated altitude (Stratosphere Hypobaric, Training Chamber) as determined from heart rate (HR), peripheral oxygen saturation (SpO_2_), and the ratio of HR response relative to hemoglobin O_2_ (oxygen) saturation in the circulating peripheral blood (HR/SpO_2_).

## 2 MATERIALS AND METHODS

### 2.1 Ethical approval and recruitment

Ethical approval for this study was granted by Mayo Clinic IRB (18‐000484). This study was conducted in accordance with the Declaration of Helsinki 2013. Healthy individuals, ranging from generally active to competitive athletes, between the ages of 18 and 80 years with history of good health were recruited. Written informed consent was obtained from all individuals prior to participation. Individuals were also required to obtain General Practitioner approval prior to participation. Individuals with history of cardiac or pulmonary disease, who were not currently active, unable to exercise or meet study requirements (e.g., number of sessions), pregnant, or live at altitude (>2100 m or 7000 ft) were excluded.

### 2.2 Study design

**FIGURE 1:**
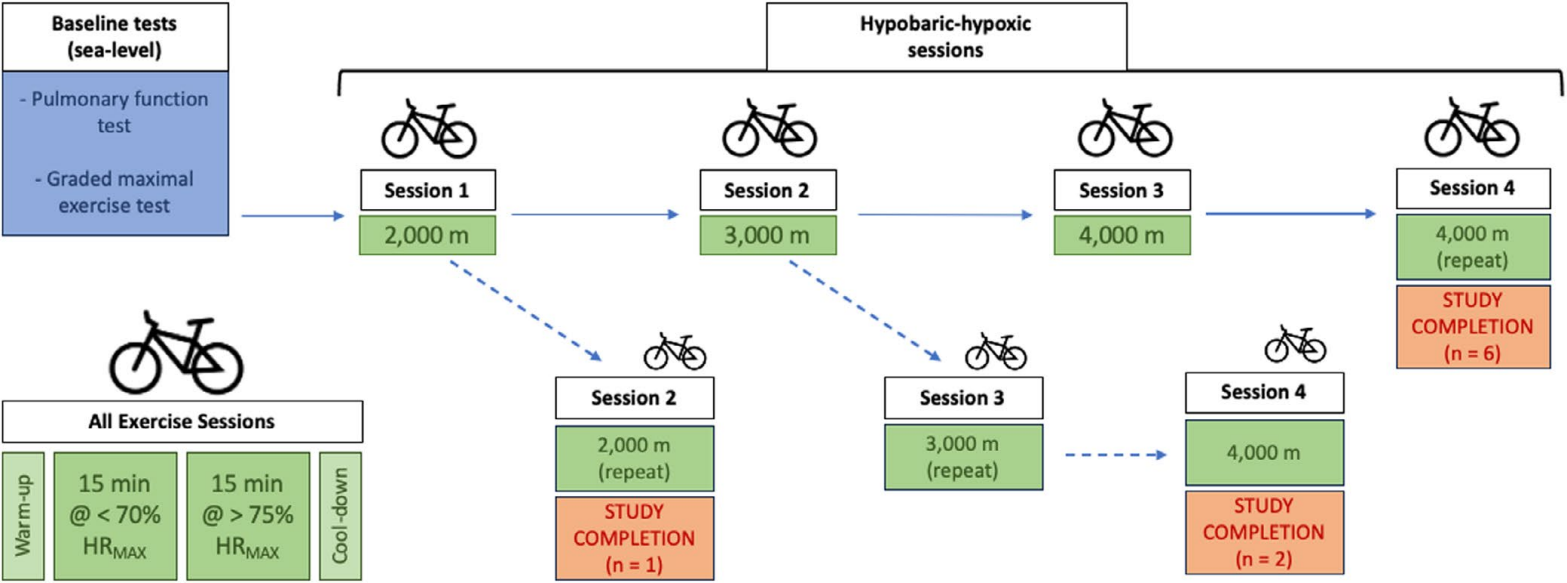
Study timeline for hypobaric hypoxic sessions. If participants could maintain >70% SpO_2_ and <70% HR_MAX_ at the target workload during the first 15 min of the session, altitude was increased for the subsequent session (solid arrow). If participants could not maintain >70% SpO_2_ and <70% HR_MAX_ at the target workload during the first 15 min of the session, the same altitude was used for the next session (dashed arrow). All exercise sessions were at least 72 h apart.

All participants completed one baseline (379 m) session and were then exposed to progressively higher simulated altitudes (across subsequent sessions) with all participants completing at least two altitude chamber (hypobaric‐hypoxic, HH) sessions as outlined next (see Figure 1).

#### 2.2.1 Baseline measurements and testing

Baseline sessions included: a physiologic tolerance test to ensure ability to safely equalize middle ear pressure (as per Mayo Clinic Hyperbaric and Altitude unit's internal protocol), a blood draw to rule out anemia, and the measurements, questionnaires, and tests (e.g., maximal exercise test, spirometry) described next.

##### Characteristics and questionnaires

Measures of height, body mass, and ambient temperature were recorded at baseline. Included individuals were also asked to complete general healthy history (Physical Activity Readiness Questionnaire, PAR‐Q) and activity (Short Form of International Physical Activity Questionnaire, IPAQ‐SF) questionnaires.

##### Lung function testing

Spirometry tests were conducted using a metabolic cart (Ultima CPX Metabolic Stress Testing System, MGC Diagnostics, St. Paul, MN) in accordance with American Thoracic Society standards (Miller et al., 2005). Participants performed maneuvers for measurements of forced vital capacity (FVC), forced expiratory volume in 1 s of FVC (FEV_1_), forced expired flow rate at 25%–75% of the FVC (FEF_25–75_), slow vital capacity (SVC), and maximum voluntary ventilation (MVV). Inspiratory capacity (IC) and tidal volume (V_T_) were also measured during spirometry tests.

##### Maximal aerobic exercise testing

Resting measurements for heart rate (HR; via 12‐lead ECG), peripheral oxygen saturation (SpO_2_, via a forehead pulse oximeter; Radical‐7, Masimo, Irvine, CA), and BP (manual sphygmomanometry) were obtained prior to the initiation of exercise tests. Participants were then fit to a cycle ergometer (Corival Lode B.V., Netherlands) and commenced exercise at 50 to 100 watts (W). Resistance was increased by 25 W per minute until maximal effort was achieved (i.e., when two out of three indications of exhaustion were observed). Indications of maximal effort/exhaustion were defined as: (1) an inability to maintain a pedal rate between 60 and 80 rpm; (2) respiratory exchange ratio (RER, >1.15); and (3) a rating of perceived exertion (RPE) ≥18 on the Borg scale (ranging from 6 to 20) (American College of Sports Medicine et al., 2018). Classic progressive protocols were followed to achieve a peak oxygen consumption (VO_2peak_) within an 8‐ to 12‐min time window. VO_2peak_ was estimated as 10 s average. Gas exchange measures included oxygen consumption (VO_2_), carbon dioxide produced (VCO_2_), breathing pattern, inspiratory timing, and minute ventilation (V_E_) were conducted using a metabolic cart (Ultima CPX Metabolic Stress Testing System, MGC Diagnostics, St. Paul, MN). Heart rate, gas exchange measures, and SpO_2_, were monitored continuously while BP, dyspnea, and RPE were recorded every 2–3 min throughout exercise tests.

#### 2.2.2 Hypobaric‐hypoxic sessions

Participants completed hypobaric‐hypoxic (HH) sessions in a hypobaric chamber (Fink Engineering, Queensland, Australia and Stratosphere Hypobaric Training Chamber, Fountain Hills, AZ, USA) during which they underwent resting physiologic measurements and a 30‐min submaximal exercise bout at altitudes dependent on individual (see next section). Ambient temperature, humidity, and altitude were recorded during these sessions. Hypobaric‐hypoxic sessions were avoided in the immediate 24‐h following any air travel or trips to altitude (e.g., Flagstaff, AZ) and were separated by at least 72 h.

##### HH exposure

The first HH session was conducted at 2000 m (6,562 ft) with altitude increased (by ~1000 m or 3281 ft; up to 4000 m) each session thereafter as allowed by individual physiologic tolerance/feedback. Individual tolerance was determined by the subject's ability to maintain variable‐dependent exercise intensity at a percentage of maximum heart rate (described next). If the participant was unable to maintain >70% SpO_2_ during the initial 15 min at the new altitude, then the altitude was reduced back to the previous session's altitude and an increased hypobaric‐hypoxic stimulus was attempted on the subsequent visit. Individuals completed at least two, and a maximum of four, HH‐sessions (refer to Figure 1).

##### HH submaximal exercise

HH exercise was performed on a cycle ergometer for 30 min (excluding warm‐up and cool‐down) continuously while maintaining SpO_2_ above 70%. Excluding the warm‐up and cool‐down, submaximal exercise was divided into two 15‐min halves, the first was conducted at light‐to‐moderate intensity (15 min, target <70% HR_MAX_), and the latter was conducted at higher intensity (target >75% HR_MAX_). Submaximal intensities were estimated from individuals' baseline VO_2peak_ results. HR and SpO_2_ were monitored continuously throughout HH sessions with HR/SpO_2_ ratio calculated from continuous data. HR/SpO_2_ ratio was utilized to assess the effectiveness of the cardiopulmonary system's ability to maintain oxygenation under stress with a value greater than one indicative of poor tolerance. RPE, dyspnea (via modified‐Borg Scale (Burdon et al., 1982)), and self‐perceived physiological altitude adaptation score (Wheatley et al., 2017) were recorded every 2–3 min.

### 2.3 Statistical analysis

Normality of distribution was assessed with outliers removed where appropriate prior to statistical analysis. Mean ± SDs were estimated for baseline measures and physiologic responses for each 15‐min half of submaximal exercise. Paired samples *t*‐tests were used to compare between consecutive HH sessions (conducted at same altitude) for physiologic responses averaged across the entire session (across both intensities within the session). Statistical significance was set at an alpha level of *p* ≤ 0.05.

## 3 RESULTS

Nine healthy, active adults (4 male, 5 female; 37.9 ± 12.7 yrs; 174.7 ± 9.4 cm; 75.3 ± 15.9 kg; and 24.4 ± 3.4 kg/m^2^) without cardiac or pulmonary disease were included, and completed at least two HH sessions at the same altitude.

### 3.1 Baseline measurements

Results for baseline spirometry and baseline maximal exercise tests are presented in Tables 1 and 2.

**TABLE 1:** Baseline spirometry results.

Baseline spirometry	
FVC (L)	4.9 ± 1.4
FVC (% pred.)	103.7 ± 10.7
FEV_1_ (L/s)	3.9 ± 1.1 L
FEV_1_ (% pred.)	101.9 ± 10.6
FEF_25–75_ (L/s)	3.9 ± 1.6
FEV_1_/FVC (%)	80.9 ± 7.7
SVC (L)	4.8 ± 1.5 L
SVC (% pred.)	110.1 ± 31.5
IC (L)	3.8 ± 1.4
IC (% pred.)	115.1 ± 25.0
MVV (L)	151.9 ± 41.7
MVV (% pred.)	106.4 ± 21.2

*Note*: Mean ± SD.

Abbreviations: FEF_25–75_, forced expiratory flow at 25%–75% of the FVC; FEV_1_, forced expiratory volume over 1 s; FVC, Forced vital capacity; IC, inspiratory capacity; MVV, maximal voluntary ventilation; RR, respiratory rate during the MVV; SVC, slow vital capacity; TV, tidal volume.

**TABLE 2:** Results for baseline physiologic measures at rest and peak/maximal exercise at sea‐level.

	Rest	Maximal/peak exercise	*p* Values
SBP (mm Hg)	116 ± 11	170 ± 19	<0.001*
DBP (mm Hg)	80 ± 11	66 ± 7	<0.001*
HR (bpm)	71 ± 18	178 ± 19*	<0.001*
% Predicted HR_MAX_	–	97 ± 8	–
SpO_2_ (%)	99 ± 1	97 ± 3*	0.042
HR/SpO_2_	0.71 ± 0.94	1.84 ± 0.17	<0.001*
VO_2_ (mL/kg/min)	4.3 ± 1.0	32.9 ± 4.1*	<0.001*
VO_2PEAK_ (% pred.)	–	102.1 ± 23.5	–
VCO_2_ (L/min)	0.3 ± 0.1	3.3 ± 0.8	<0.001*
V_E_ (L/min)	12.4 ± 4.0	104.2 ± 24.5*	<0.001*
W_MAX_ (watts)	–	225 ± 57	–
RER	1.00 ± 0.07	1.32 ± 0.09	<0.001*
RPE	–	17 ± 1	–

*Note*: Paired *t*‐tests were used to compare between resting and peak exercise for physiologic measurements. All statistical tests were two‐tailed with significance set to alpha <0.05 and (*) used to denote significant difference compared to resting measurements. Mean ± SD.

Abbreviations: DBP, diastolic blood pressure; HR, heart rate; RER, respiratory exchange ratio; RPE, rating of perceived exertion; SBP, systolic blood pressure; SpO_2_, peripheral blood oxygen saturation; VCO_2_, volume of carbon dioxide; V_E_, minute ventilation; V_E_/VCO_2_, ventilatory equivalent for carbon dioxide; VO_2_, oxygen consumption; VO_2PEAK_, maximal oxygen consumption; W_MAX_, maximal power output in watts.

### 3.2 Hypobaric‐hypoxic (HH) sessions

Nine participants completed repeat HH sessions at the same altitude (2000 m, *n* = 1; 3000 m *n* = 2; 4000 m, *n* = 6). Results for environmental conditions and physiologic responses from these repeat HH exercise sessions are presented in Table 3. Repeat sessions were separated by 10 ± 8 days.

**TABLE 3:** Physiologic responses for hypobaric‐hypoxic (HH) exercise sessions.

	HH visit 1		HH visit 2	
	<70% HR_MAX_	>75% HR_MAX_	<70% HR_MAX_	>75% HR_MAX_
Temperature (°C)	22.9 ± 3.8	23.2 ± 3.5	23.1 ± 4.2	23.9 ± 4.3
Humidity (%)	40.7 ± 43.2	41.6 ± 8.2	35.0 ± 9.2	39.8 ± 11.6
Mean HR (bpm)*	146 ± 18	155 ± 18	122 ± 22	143 ± 29
HR_MAX_ (bpm)*	161 ± 16	169 ± 18	137 ± 23	154 ± 30
%HR_MAX_*	82.2 ± 8.2	87.4 ± 6.8	68.4 ± 5.9	80.2 ± 10.7
SpO_2_ (%)	85 ± 5	85 ± 4	86 ± 4	84 ± 5
SpO_2MIN_	79 ± 5	80 ± 5	79 ± 6	78 ± 5
HR/SpO_2_*	1.69 ± 0.18	1.83 ± 0.24	1.43 ± 0.27	1.71 ± 0.33
Watts (W)*	89 ± 33	89 ± 40	92 ± 35	118 ± 34
% W_MAX_*	40 ± 9	39 ± 15	40 ± 10	52 ± 9
RPM	72 ± 6	74 ± 9	80 ± 25	75 ± 12
Signs & symptoms	0.6 ± 1.0	0.8 ± 1.3	0.2 ± 0.7	0.2 ± 0.7
RPE*	14 ± 2	15 ± 1	11 ± 3	12 ± 4
Dyspnea	3.8 ± 1.3	4.9 ± 1.5	2.7 ± 2.4	3.4 ± 2.8

*Note*: Paired *t*‐test was performed between the two sessions for each exercise response variable using averages of measurements from both intensities (within a session) with significant differences between the two sessions denoted by (*). All statistical tests we two‐tailed with alpha set to <0.05.

Abbreviations: DBP, diastolic blood pressure; HR, heart rate; RPE, rating of perceived exertion; RPM, rotations per minute; SBP, systolic blood pressure; SpO_2_, peripheral oxygen saturation; SpO_2MIN_, minimum oxygen saturation; VCO_2_, volume of carbon dioxide; V_E_, minute ventilation; V_E_/VCO_2_, ventilatory equivalent for carbon dioxide; VO_2_, volume of oxygen consumption; W_MAX_, maximal power output in watts.

There were no significant changes in average SpO_2_ (*p* = 0.68) or SpO_2MIN_ (*p* = 0.48) between the two visits. The average workload across both intensity levels was 89 ± 36 W for visit 1 and 105 ± 36 W for visit 2 (*p* = 0.024) while average SpO_2_ across both intensity levels was 85 ± 4 for visit and 85 ± 4% for visit 2 (*p* = 0.684). A significant difference was observed between repeat sessions for mean HR (*p* = 0.001) with an average decrease in mean HR of 18 bpm (11%) from visit 1 (150 ± 18 bpm) to visit 2 (133 ± 27 bpm). Similarly, a significant reduction in HR/SpO_2_ (*p* = 0.012) was also observed between repeat sessions with an 11% average reduction in HR/SpO_2_ from visit 1 (1.76 ± 0.22) to visit 2 (1.57 ± 0.33). A significant reduction in RPE (*p* = 0.017) was also observed from visit 1 (15 ± 2) to visit 2 (11 ± 4), being reduced on average by 3 RPE. Finally, dyspnea scores were no different between repeat sessions (visit 1: 4.4 ± 1.5 vs. visit 2: 3.1 ± 2.6, *p* = 0.19), nor were self‐perceived physiological altitude adaptation signs and symptoms scores (visit 1: 0.7 ± 1.1 vs. visit 2: 0.2 ± 0.7, *p* = 0.27) with headache being the most prominent symptom in both sessions.

## 4 DISCUSSION

The objective of this study was to evaluate physiologic response(s) to repeated HH aerobic exercise sessions and examine for evidence of physiologic adaptation. Findings from this study indicate that short aerobic exercise bouts (~30 min) performed under HH conditions are sufficient to elicit an adaptive response, which is potentially sustained more than a week following the initial session. This was most evident in the significantly higher workload maintained in visit 2 at a lower RPE, HR, and HR/SpO_2_ compared to visit 1. These observations are consistent with studies investigating single bouts of intermittent hypoxia (Collins & Solomon, 2019) and hypoxic exercise (Mackenzie et al., 2011), which have demonstrated adaptations following a single bout of hypoxic exposure (e.g., improved insulin resistance). By contrast, other studies have shown that single bouts of hypoxic exercise are insufficient to produce adaptation(s) (Slivka et al., 2014). However, these conflicting studies were predominantly intermittent hypoxic exposures (i.e., hypoxia interspersed with room air), which is inconsistent with the protocol used in the present study. Nevertheless, the observed adaptive responses appear unrelated to age, sex, or fitness level, albeit whether they are linked to oxygen sensing, neurally mediated, or indicative of early adjustments in oxygen delivery pathways is unclear.

Given the limited total hypoxic stimulus, it is unlikely that the observed improvements in exercise performance were attributed to hematological factors (e.g., increased erythropoietin and erythrocyte number and size), which occur in response to hypoxic exercise training programs (Ploszczyca et al., 2018) and altitude exposure. Rather, the observed adaptations were more likely neurally‐mediated (Bourdillon et al., 2023), or potentially redox‐related (Møller et al., 2001). It is plausible that these repeat sessions induced breaks in DNA strands (Møller et al., 2001), product of the increased reactive oxygen species (ROS) production, greater oxidative stress associated, and altered redox homeostasis, which occur in response to hypoxic exercise (Maciejczyk et al., 2022). Further, the redox status following the initial HH session(s), subsequent repair of DNA breaks, and the time course of such, may impact physiologic signaling, which in this case, may have presented as physiologic adaptation to hypoxic exercise (Radak et al., 2013) (Quindry et al., 2016). Unfortunately, however, no factors directly related to this mechanistic theory were measured in the present study and therefore, other hypotheses must also be considered.

Lower HR (e.g., visit 2 compared to visit 1) is associated with reduced cardiac output, which would be expected to improve oxygen transit time (and increase oxygen saturation). However, in the present study, the lower HR in visit 2 was observed in the absence of any significant improvement in SpO_2_. It could therefore be suggested that the reduction in HR during visit 2 was product of a reduction in cardiac sympathetic activation that has been observed to follow intermittent hypoxia (Haider et al., 2009). This reduction in sympathetic activation may potentially due to modulation of the carotid baroreceptor reflex (Iellamo et al., 1997) (Bourdillon et al., 2023), which has been shown to exhibit rapid adaptation (Potts & Mitchell, 1998). Consistent with this hypothesis, additional studies that have shown a higher number of motor units recruited during hypoxic exercise due to the greater metabolic load (Schoenfeld, 2013) and increased neural drive (in muscle) (Manimmanakorn et al., 2013). This is further supported by participants in the present study being able to maintain a higher workload in visit 2 compared to visit 1.

### 4.1 Limitations and future directions

One limiting factor of the current study was the small cohort; however, findings are still meaningful with the data providing a foundation for future studies. Unfortunately, no measurements of hypoxic chemosensitivity or baroreflex were obtained, and therefore, such mechanisms can only be speculated in the discussion. Similarly, no peripheral blood samples were collected and therefore, we cannot definitively identify any mechanistic cause for the observed results.

We acknowledge that the study design was atypical, with repeat sessions occurring at different altitudes across participants. However, given the well‐established individual variability in response to hypoxia, we contend that this design was more effective in delivering a consistent challenging hypoxic stimulus across participants. As such, we believe it was better suited for the objectives of this study. Moreover, the observed outcomes were consistent across participants, irrespective of the specific altitude and number of total altitude exposures; individuals simply reached their threshold for this response at different physiological points or degree of hypoxic stimulus. Lastly, while we recognize that the absence of a dedicated hypobaric hypoxia (HH) familiarization session means that an emotional component cannot be entirely ruled out as a factor in the differences observed between sessions, we consider this unlikely. This is because the majority (*n* = 6) showed improvement in repeat sessions, following two prior exposures, during which any substantial emotional influence would likely have manifested.

Despite these limitations, this study is important given that a single or few hypoxic exposures may have clinical implications, such as, the improved insulin sensitivity observed following single intermittent exercise sessions in hypoxia observed among type 2 diabetics (Mackenzie et al., 2012). Therefore, it is anticipated that this study will lead to further investigation of application intermittent hypobaric hypoxia exposure to human health and illness treatment with one potential research area being that of investigating the health of individuals with sleep apnea, cardiac, and metabolic diseases in clinical populations. Similarly, there is potential for application of similar future research with the Department of Defense, to improve and, potentially expedite, acclimatization among individuals who are being rapidly deployed to altitude. As such, future studies may aim to focus on redox‐sensitive pathways or neurally‐mediated factors when investigating the mechanisms for improvement exercise tolerance following a single bout of hypoxic exercise.

## 5 CONCLUSION

These findings indicate an adaptive response to a short aerobic exercise bout during simulated altitude at an individual's hypoxic threshold exposure that appears to be sustained greater than 1 week after the initial visit. Whether this is linked to oxygen sensing, neurally mediated or suggests early adjustments in oxygen delivery pathways is unclear, and thus, further investigations are required.

## AUTHOR CONTRIBUTIONS

K.E.J.: formal analysis (lead); writing—original draft (lead); writing—review and editing (equal); and visualization (lead). M.T.B.: formal analysis (supporting); writing—original draft (supporting); and writing—review and editing (equal). C.M.W‐G: conceptualization and design (lead); investigation (equal); data curation (equal); investigation (equal); writing—review and editing (equal); visualization (supporting); supervision (lead); and project administration (lead). J.C.S. and J.K.P.: investigation (equal); data curation (equal); and writing—review and editing (equal). B.D.J.: conceptualization and design (supporting); writing—review and editing (equal); and project administration (supporting).

## FUNDING INFORMATION

This study was supported by Mayo Clinic Arizona Cardiovascular Research Center Clinical Research Grant (MCA CV CRC), Bruce D. Johnson, Flinn Foundation, Bruce D. Johnson, ENSELE, Courtney M Wheatley‐Guy, Innovation in Aging Award from the Mayo Clinic Center for Clinical and Translational Science (CCaTS) and the Robert and Arlene Kogod Center on Aging, Courtney M Wheatley‐Guy. We are thankful for their generous support. Contents of this publication are solely the responsibility of the authors and do not necessarily represent the official views of the MCA CV CRC, FLINN Foundation, ENSELE or CCaTS.

## CONFLICT OF INTEREST STATEMENT

No competing financial interests exist.

## Data Availability

Data that support these findings are available upon reasonable request from the corresponding author. Data are not publicly available for privacy reasons.
